# Supplementary material for: 5′UTR G-quadruplex structure enhances translation in size dependent manner
Source: Nat Commun. 2024 May 10;15:3963. doi: 10.1038/s41467-024-48247-8 (PMC11087576; doi:10.1038/s41467-024-48247-8)
Supplement: Supplementary file 1 — Supplementary Information [file 41467_2024_48247_MOESM1_ESM.pdf]

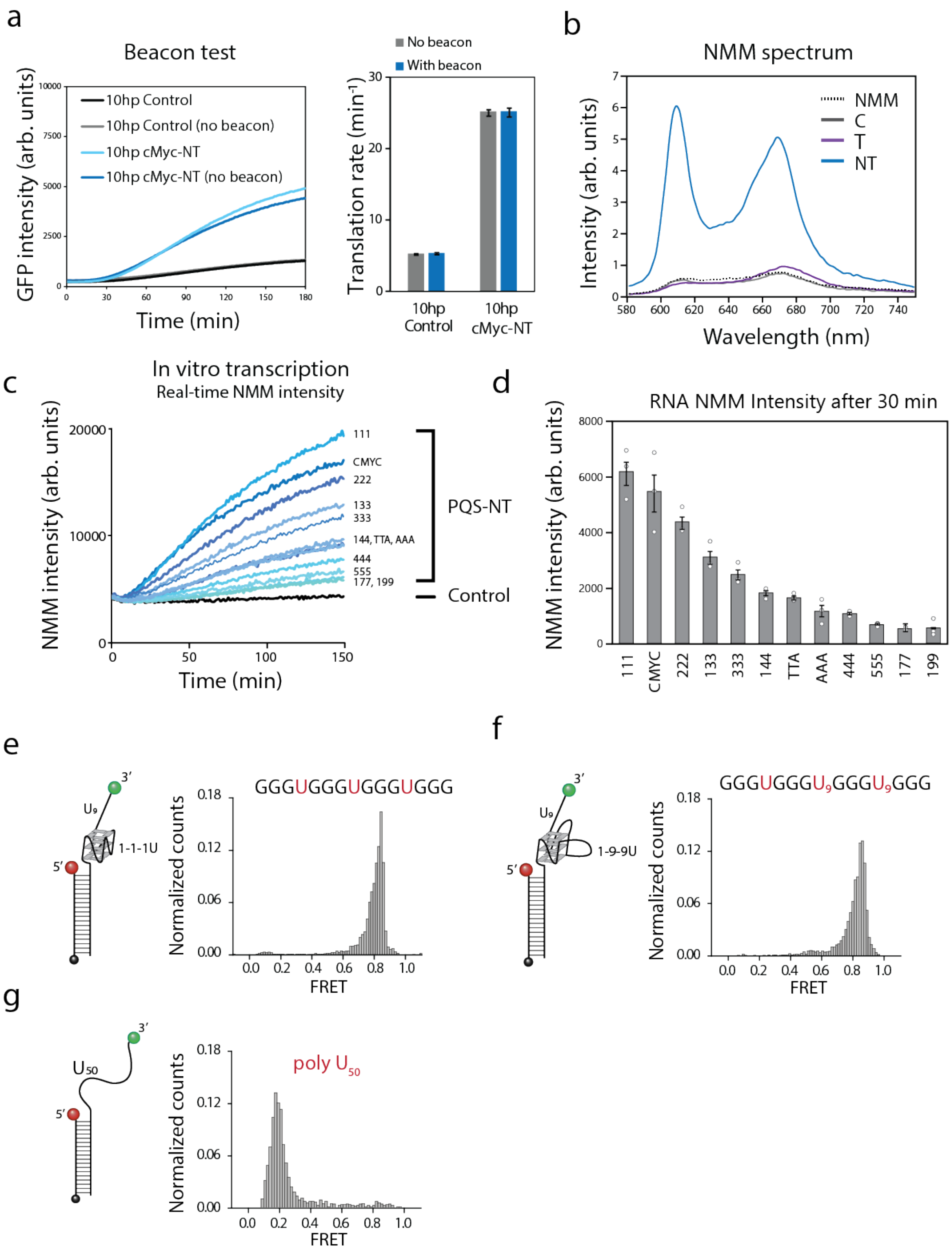

Supplementary Fig. 1: Beacon effect verification, and RNA G-quadruplex formation verified by G4-binding ligand and smFRET.

a, Molecular beacon shows no impact on translation rate. The curves shown are one representative result from two independent experiments. The bar graph was plotted by the fitted results of the curves with 95% confidence interval. **b-d,** RNA G4 formation is examined by NMM, which selectively binds to parallel G4. **b,** Fluorescence spectrum of NMM excited at λ_{ex} 393 nm. CMYC-NT RNA shows a clear emission peak at λ_{em} 610 and 670 nm, indicating strong binding of NMM. **c,** Real-time NMM fluorescence detected in the *in vitro* transcription assay. All PQS-NT show an increase of NMM intensities, demonstrating the formation of RNA G4. The curves shown are one representative result from multiple independent experiments. **d,** The NMM intensities of all PQS-NT constructs at the 30 min time point. The intensity trend is correlated to the size of PQS (shown in **Figure 3a**). Data are presented as mean ± SEM of n = 3 independent experiments. The exact mean value and raw data points are provided as a **Source Data** file. **e, f** RNA G4 formation detected by smFRET assay. **e,** smFRET test of “111” RNA. **f,** smFRET test of “199” RNA. Both RG4 constructs show a steady FRET efficiency without dynamics, indicating both small and bulky RG4 stably folded. **f,** smFRET test of poly U_{50} RNA in representative of unfolded RNA state.

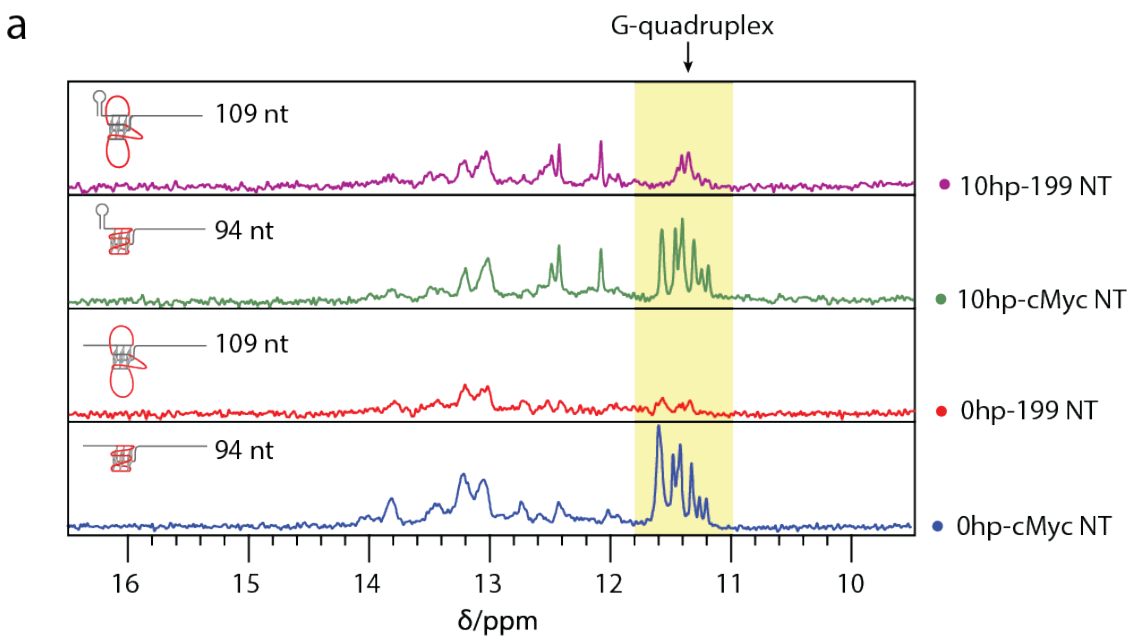

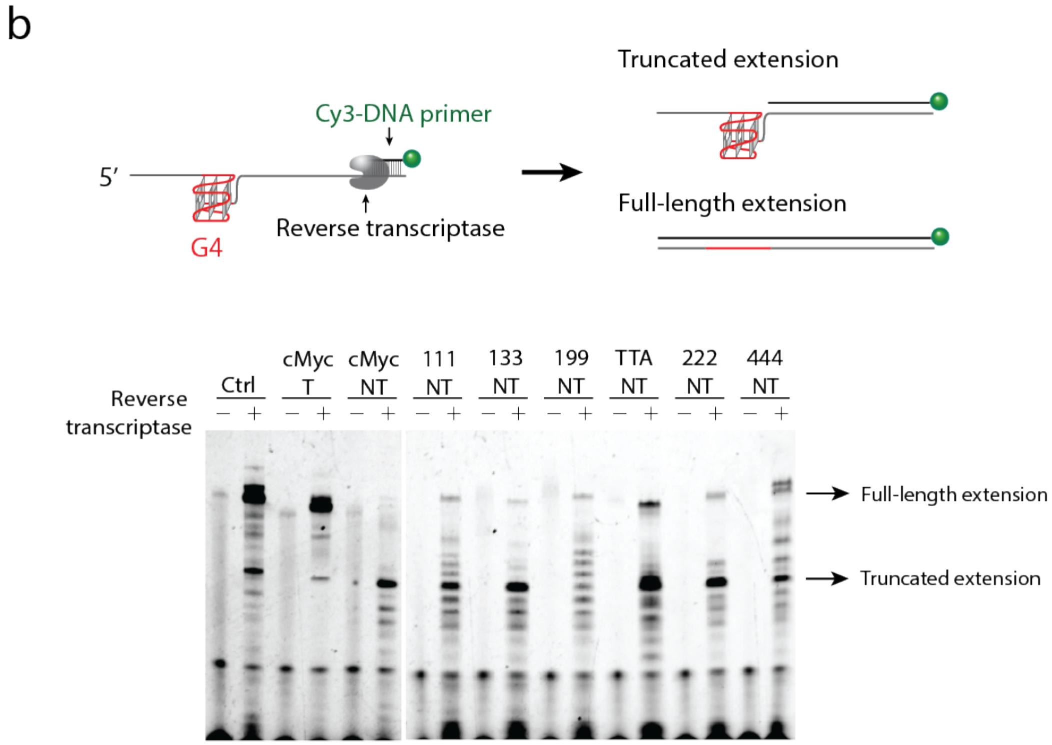

Supplementary Fig. 2: Varying sizes of RG4 fold into G-quadruplex structure.

a, RNA G-quadruplex structures verified by NMR. The graph shows part of the 1-D ^{1}H spectrum of four RG4-containing RNAs. The G4 region (indicated in yellow block) represents the chemical shift of imino protons within G-quadruplex structure. The RNA molecules are 5'UTR of 0hp-cMyc-NT (blue), 0hp-199-NT (red), 10hp-cMyc-NT (green), and 10hp-199 NT (purple). The length of cMyc containing RNA is 94 bases, and 199 containing RNA are 109 bases. Both cMyc-NT RNA show strong G4 signals with sharp peaks. The presence of G4 imino peaks in the 199- NT RNA signifies G4 formation, with G4 in 10hp 199-NT being more stable than 0hp 199-NT. **b,** Biological stability of RG4 examined by reverse transcriptase stop assay. The formation of stable RNA structure results in truncated cDNA by stopping the polymerization of reverse transcriptase. All the RG4-containing RNAs show truncated cDNA bands while Ctrl and cMyc-T show strong band of full-length cDNA, indicating all the RG4 has relative stable structures that stopped the polymerization. The image shown is one representative result from two independent experiments.

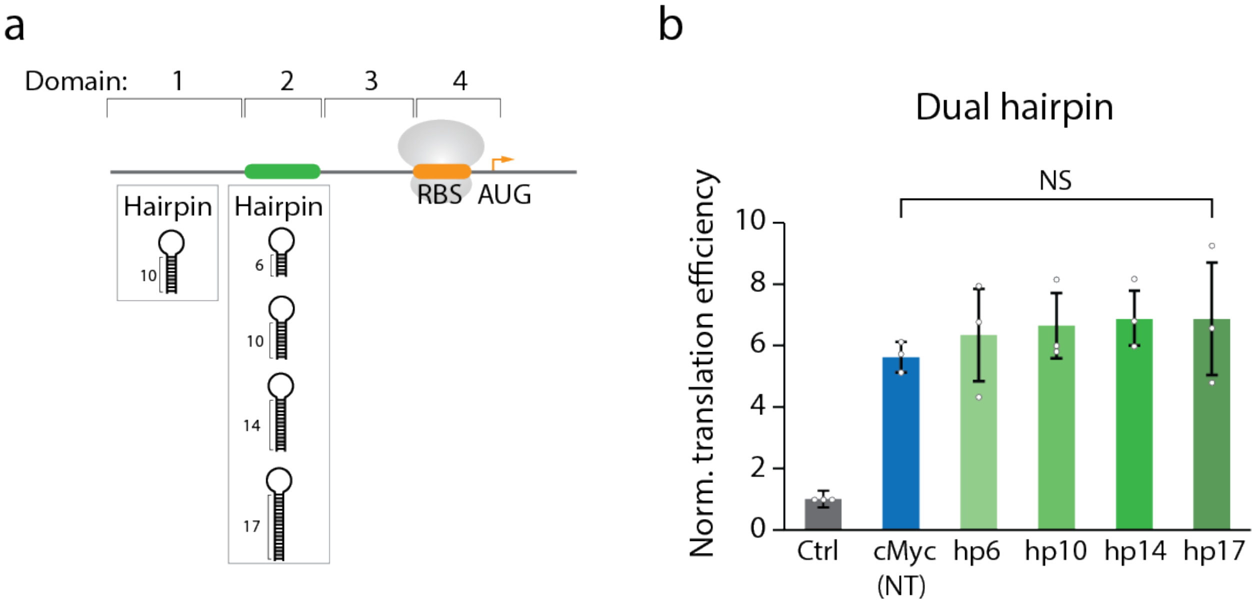

Supplementary Fig. 3: Dual hairpin structures cause the same level of enhancement.

a, Schematic of the dual hairpin constructs. The first hairpin is fixed to 10 hp. The second hairpin is inserted at domain 2 (green) to replace the PQS with either 6, 10, 14, or 17 bp stem hairpin. The folding energy is calculated by UNAfold and listed in **Supplementary Table 1**. **b,** Normalized translation efficiency of dual hairpin constructs. The data are presented as mean ± SEM of independent experiments (n = 3). NS: non-significant (two-sided paired t-test). Exact mean values are provided in **Supplementary Tables 3.11**. Raw data points are provided as a **Source Data** file.

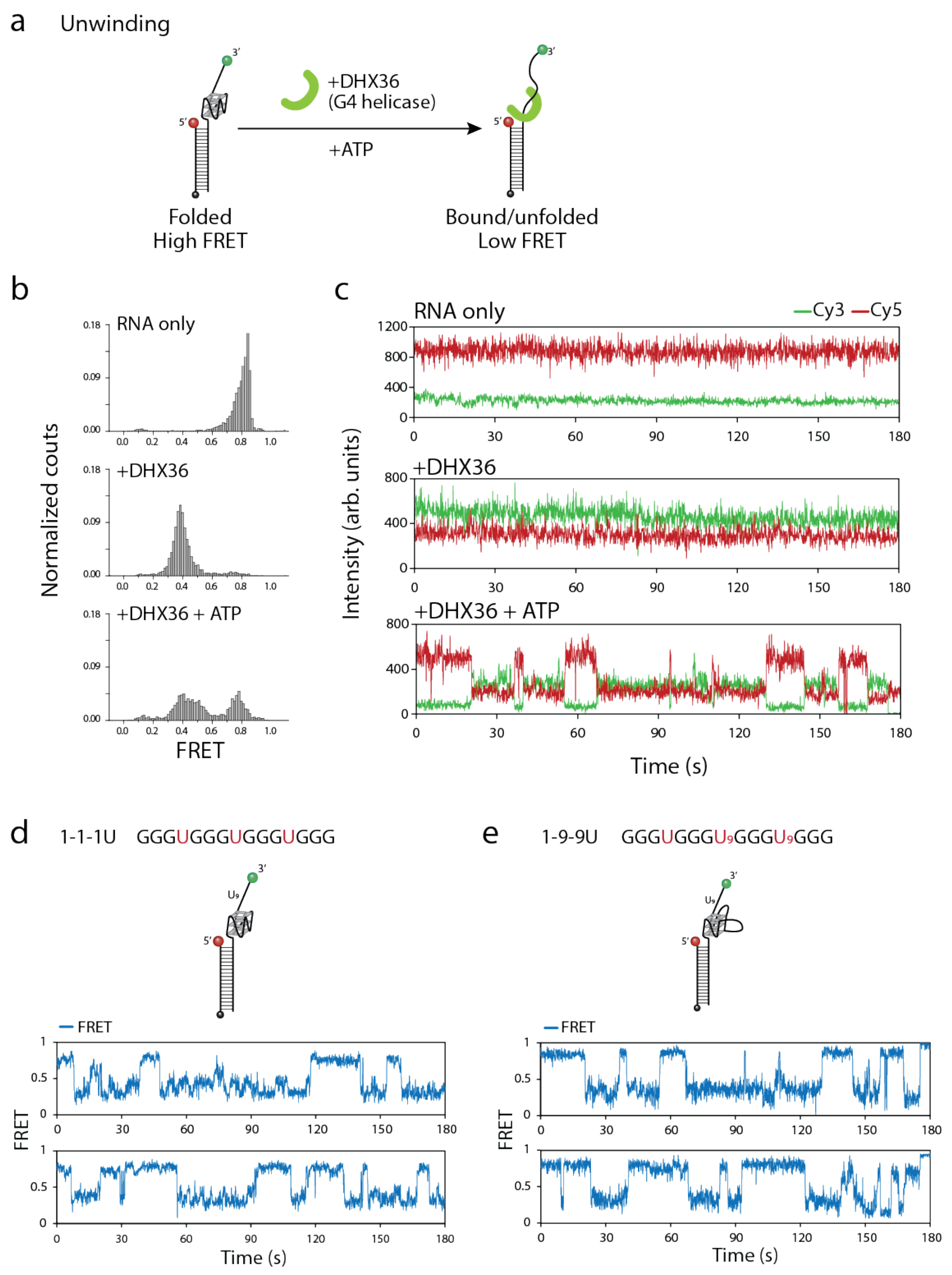

Supplementary Fig. 4: DHX36 helicase unwinds both tight and bulky RG4.

a, Schematic of smFRET RG4 unwinding assay. **b,** FRET efficiency shifts to low FRET after adding DHX36 helicase, indicating the binding of helicase. DHX36 and ATP together demonstrate broad FRET states, suggesting dynamics of FRET transition. **c,** In the presence of ATP, the repeated anti-correlation of fluorescence represents FRET transition, providing the evidence of repeated unwinding and re-folding process. **d, e,** Examples of FRET traces showing unwinding events of both 111 (tight) and 199 (bulky) RG4 in the presence of DHX36 and ATP.

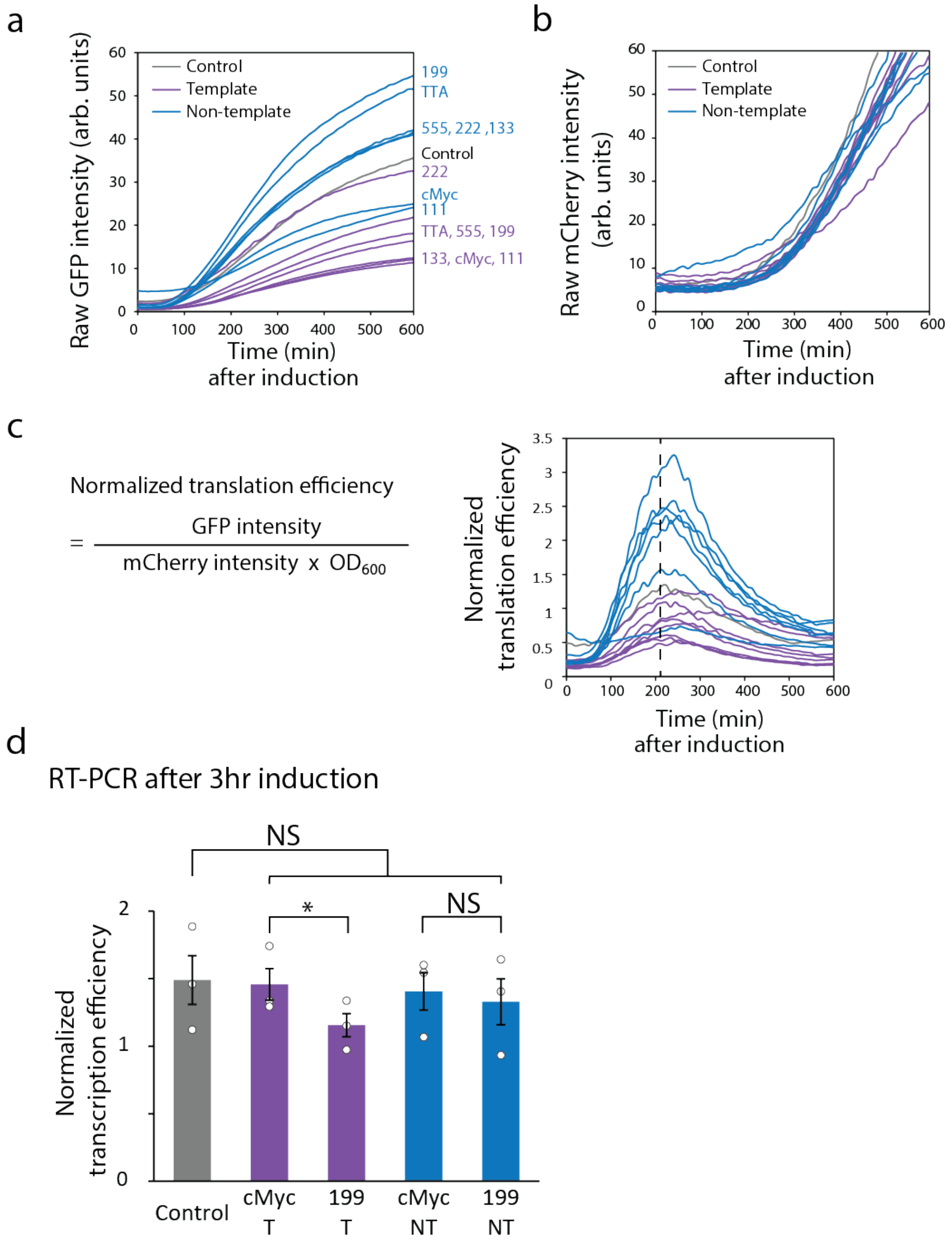

Normalized transcription efficiency

$$= \left(\mathrm{Cq}_{\mathrm{mCherry}} - \mathrm{Cq}_{16\mathrm{S\ rRNA}}\right) - \left(\mathrm{Cq}_{\mathrm{GFP}} - \mathrm{Cq}_{16\mathrm{S\ rRNA}}\right) = log_2 \frac{[\mathrm{GFP\ mRNA}]}{[\mathrm{mCherry\ mRNA}]}$$

Supplementary Fig. 5: Quantification of mRNA and reporter expression.

a, b, Real-time fluorescence of GFP and mCherry detected by plate reader. The orientation dependence is observed in GFP expression due to the insertion of PQS while mCherry expression shows no difference among all the strains. **c,** The translation efficiency of GFP is normalized by the cell density and mCherry as an internal control. The efficiency varies by time because of the different maturation time between two reporters. The efficiency at 210 min time point (dash line) is defined as highest expression level and plotted in **Figure 7e**. All the curves shown are a representative result from three independent experiments. **d,** mRNA amount between control, template, and non-template construct shows no significant difference, indicating the enhancement is mainly induced at translation process. The data are presented as mean ± SEM of independent experiments (n = 3). NS: non-significant, *P < 0.05 (two-sided paired t-test). Exact values are provided in **Supplementary Tables 3.12**. Raw data points are provided as a **Source Data** file.

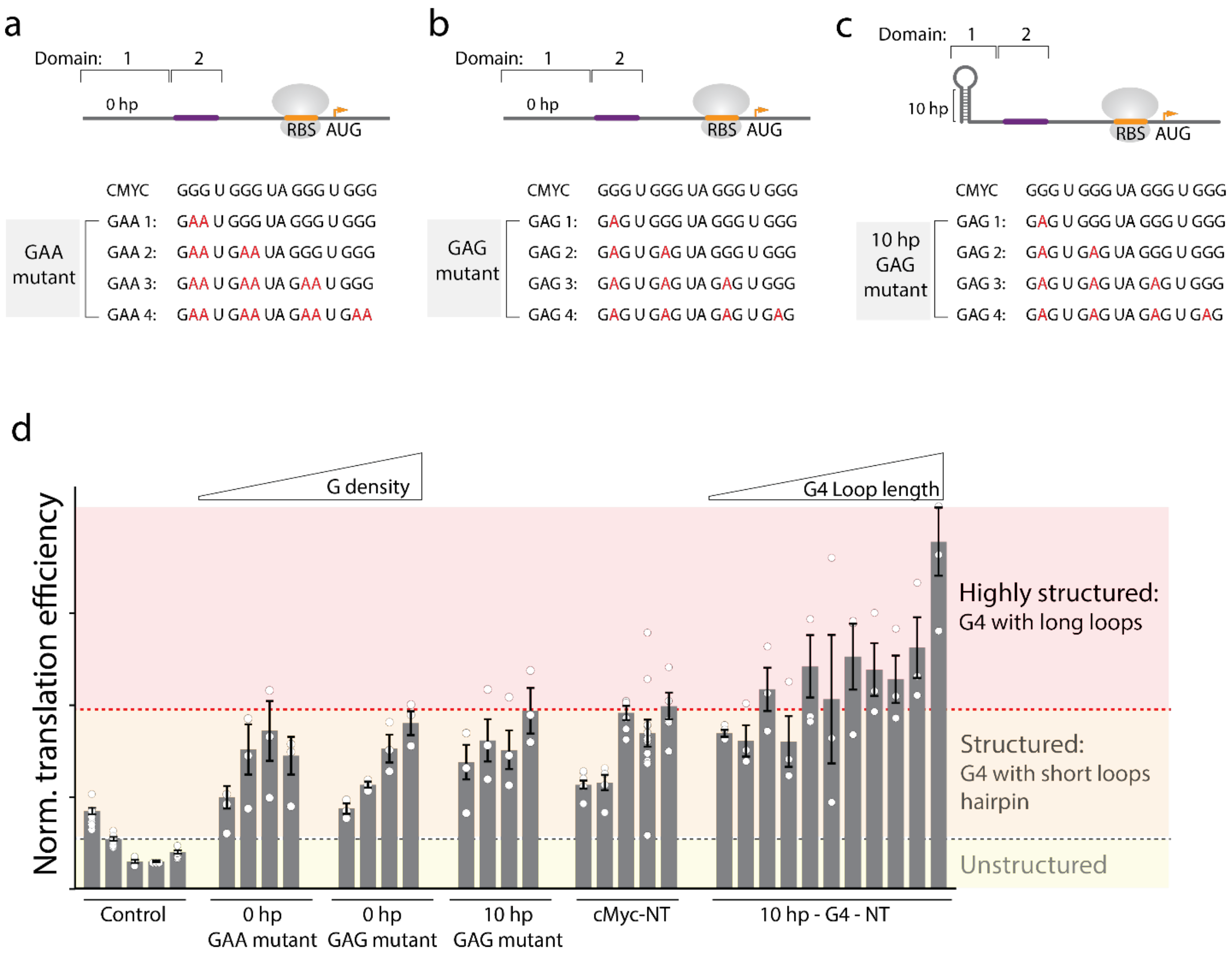

Supplementary Fig. 6: Mutant G4s have similar effect on translation as CMYC.

a, b, c, Schematic of mutant CMYC constructs. The first hairpin is fixed to 10 hp in **c** and removed in **a** and **b**. GAG mutant means guanine in the second tetrad is mutated stepwise into adenine at domain 2 (purple), and GAA mutant means guanines in both second and third are mutated into adenine. The folding energy is calculated by UNAfold and listed in **Supplementary Table 1**. **d,** Summary of translation efficiency. Translation efficiency of 0hp-GAA mutants, 0hp-GAG mutants, 10hp-GAG mutants are quantified and performed statistical tests are provided in **Supplementary Table 3-13, 3-14, and 3-15**. The data are presented as mean ± SEM of independent experiments (n = 3). Raw data points are provided as a **Source Data** file. Part of the graph was combined with selected data from **Figure 4b**, which are control (0hp to 12hp), cMyc-NT (0hp to 12hp), and other 10hp-G4-NT. The graph demonstrates the unstructured controls have lowers translation efficiency, short loop G4 and its mutants have medium level of translation, and a G4 loop length dependence.

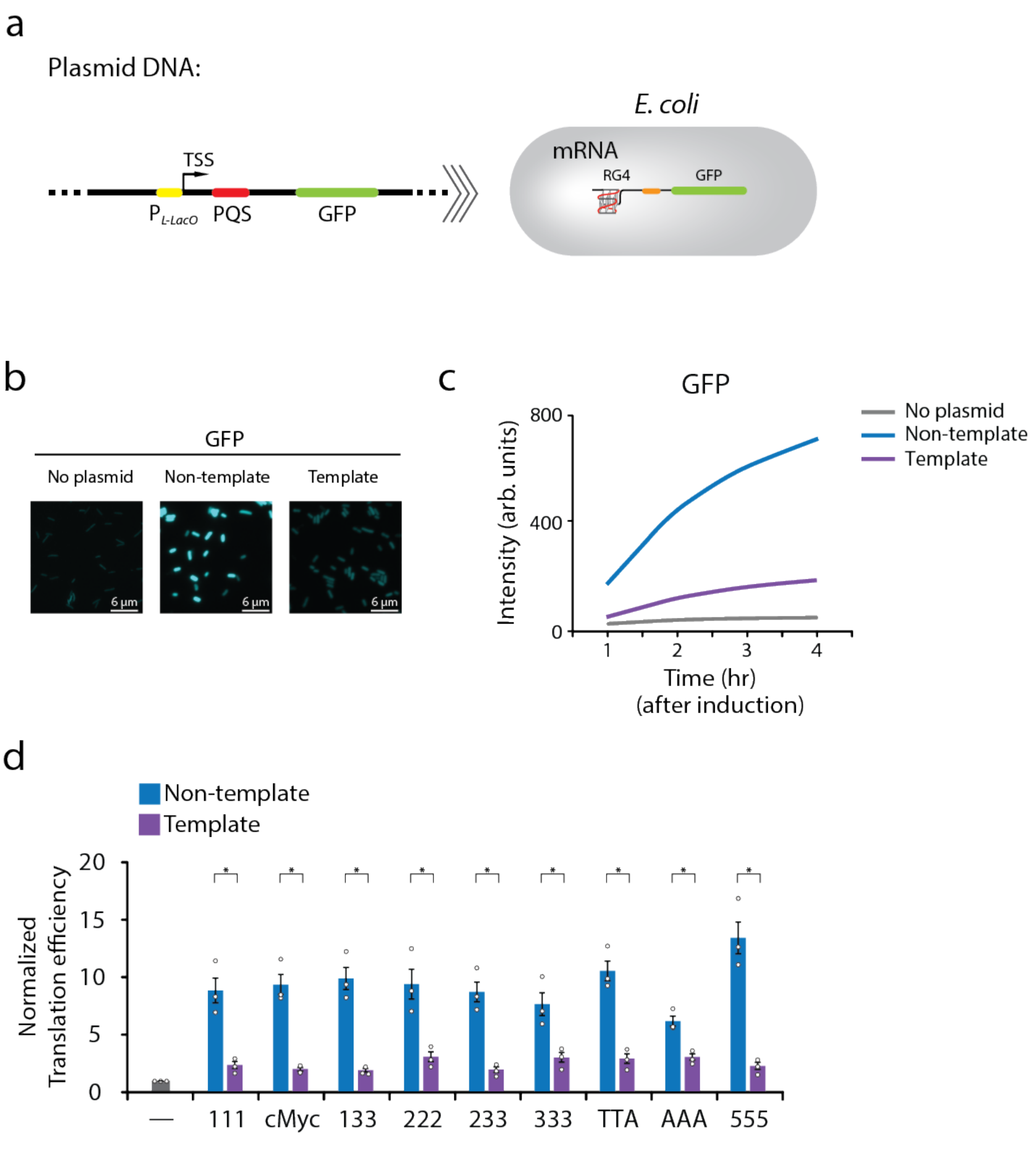

Supplementary Fig. 7: Orientation-dependence observed in *E. coli* promoter.

a, Schematic of PQS constructs with *E. coli* promoter $P_{L\text{-}LacO}$. **b,** Fluorescence imaging of GFP expression in *E. coli*. The scale bar indicates 6 µm (50 px). The images shown are one representative result from n = 3 independent experiments. **c,** Real-time GFP intensities. The curves represent example traces of cMyc, where non-template (NT) and template (T) are colored

in blue and purple, respectively. The data is collected by plate reader after IPTG induction. The curves shown are one representative result from n = 3 independent experiments. **d,** Translation efficiencies demonstrate the orientation dependence between NT and T. Data are presented as mean ± SEM of n = 3 independent experiments. Shown in **d** only represents the significance between template and non-template, where *$P < 0.05$ (two-sided paired t-test). Exact mean values are provided in **Supplementary Tables 3.16**. Raw data points are provided as a **Source Data** file.

Supplementary Table 1: RNA folding energy prediction.

Domain	Name	Sequence (5' → 3')	*ΔG* (kcal/mol)
1	0 hp	GGACAAUUGUGAGCGUGUAGUCAGUGAUUUGAUCAGAAUUCUUU	-3.1
	4 hp	GGACAA**UUGU**GAGCGGAUA**ACAA**GUGAUUUGAUCAGAAUUCUUU	-5.9
	8 hp	GG**GGAAUUGU**GAGCGGAUA**ACAAUUCC**AUUGAUCAGAAUUCUUU	-10.4
	10 hp	**GGGGAAUUGU**GAGCGGAUA**ACAAUUCCCC**UCUAGAGAAUUCUUU	-15.9
	12 hp	**GGGGAACGUUGU**GAGCGGAUA**ACAACGUUCCCC**UAGAAUUCUUU	-21.4
3		UUACCGGUUUUGGUUGAAGGUAGUGGUAGUGGUGAUAUCCUGC	-5.6
4		AGGAAAGGAGAGUAGCAAUGGGUACC	0.9
3+4		UUACCGGUUUUGGUUGAAGGUAGUGGUAGUGGUGAUAUCCUGC<br>AGGAAAGGAGAGUAGCAAUGGGUACC	-11.8
2	6 hp	**UCGAAC**UUUU**GUUCGA**	-6.4
	10 hp	**GCAGCGAGGC**UUUUU**GCCUCGCUGC**	-21
	14 hp	**GACGGCAGCGAGG**CUUUUU**GCCUCGCUGCCGUC**	-31.3
	17 hp	**GGCGACGGCAGCGAGGC**UUUUU**GCCUCGCUGCCGUCGCCA**	-42.1
	GAG 1	GAG U GGG UA GGG U GGG	2.4
	GAG 2	GAG U GAG UA GGG U GGG	4.6
	GAG 3	GAG U GAG UA GAG U GGG	4.6
	GAG 4	GAG U GAG UA GAG U GAG	4.6
	GAA 1	GAA U GGG UA GGG U GGG	2.4
	GAA 2	GAA U GAA UA GGG U GGG	3.7
	GAA 3	GAA U GAA UA GAA U GGG	2.9
	GAA 4	GAA U GAA UA GAA U GAA	2.9

* The folding energy was calculated by UNAFold (Source: http://www.unafold.org/mfold/applications/rna-folding-form.php).

** Bold letters indicate the predicted hairpin stem.

Supplementary Table 2: Sequences of oligos in each assay.

DNA oligo for PCR	
T7 forward primer	5'- GCT TTC GTC TTC ACC TCG AG -3'
T7 reverse primer	5'- AAA CCC CTC CGT TTA GAG AGG GGT TAT GCT AG TTA TTT GTA GAG CTC ATC CAT GCC ATG -3'
Short-length primer 1 (reverse)	5'- ACC AAC TTC CAT CAC CAT CAC CAC -3'
Long-length primer 2 (reverse)	5'- GTT CTT CTC CTT TGC TGG TAC -3'
Reverse transcription primer	5'- Cy3/GTT CTT CTC CTT TGC TGG TAC -3'
Molecular beacons	
Cy3-Probe 3	5'- Cy3/TTC ACC CTC TCC ACG GAC/3BHQ_2/ -3'
RBS probe	5'- Cy5/GCT ACT CTC CTT TCC TGC A/3BHQ_2/ -3'
qPCR primers	
qPCR Primer 1 forward	5'- AGA GTA GCA ATG GGT ACC AGC -3'
qPCR Primer 1 reverse	5'- AAA ATT TGT GCC CAT TAA CAT CAC C -3'
qPCR Primer 2 forward	5'- GAT GGC CCT GTC CTT TTA CCA -3'
qPCR Primer 2 reverse	5'- CAT GTG GTC ACG CTT TTC GT-3'
mCherry qPCR forward primer	5'- CAC TAC GAC GCT GAG GTC AA -3'
mCherry qPCR reverse primer	5'- TAG TCC TCG TTG TGG GAG GT-3'
GFP qPCR forward primer	5′- GAT GGC CCT GTC CTT TTA CCA -3′
GFP qPCR reverse primer	5'- CAT GTG GTC ACG CTT TTC GT-3'
RNA samples (HPLC purification)	
Poly U_{40} RNA	5'- rU_{40}-3'
cMyc RNA	5'-rUrCrA rUrArG rGrGrU rGrGrG rUrArG rGrGrU rGrGrG rCrUrG rGrA-3'
Biotin 18mer	5'-/5AmMC6/rGrCrC rUrCrG rCrUrG rCrCrG rUrCrG rCrCrA/3Bio/-3'
111-U9 RNA	5′-rUrGrG rCrGrA rCrGrG rCrArG rCrGrA rGrGrC rUrUrG rGrGrU rGrGrG rUrGrG rGrUrG rGrGrU rUrUrU rUrUrU rUrU /3AmMO/-3′
199-U9 RNA	5′-rUrGrG rCrGrA rCrGrG rCrArG rCrGrA rGrGrC rUrUrG rGrGrU rGrGrG rUrUrU rUrUrU rUrUrU rGrGrG rUrUrU rUrUrU rUrUrU rGrGrG rUrUrU rUrUrU rUrUrU/3AmMO/-3′
Poly U_{50} RNA	5′-rUrGrG rCrGrA rCrGrG rCrArG rCrGrA rGrGrC rU_{50} /3AmMO/-3′

* All the oligos were purchased from Integrated DNA Technologies (IDT)

Supplementary Table 3: Statistics Result

Supplementary Table 3.1: Normalized transcription rate (Fig. 2c)

				P-value	
Name	Mean	SEM	N value	Control	Template
Control	1.00	0.06	43		
Template	0.87	0.09	6	0.034	
Non-template	1.16	0.12	12	0.009	0.012

*N value means the number of independent events.
*SEM is presented as 95% confidence limit.
**P*-values are reported by two-sided unpaired t-test.

Supplementary Table 3.2: Normalized translation efficiency (Fig. 2e)

				P-value	
Name	Mean	SEM	N value	Control	Template
Control	1.00	0.03	14		
Template	0.90	0.23	6	0.294	
Non-template	5.73	1.02	12	5.95366E-05	4.08359E-05

*N value means the number of independent events.
*SEM is presented as 95% confidence limit.
**P*-values are reported by two-sided unpaired t-test.

Supplementary Table 3.3: Normalized translation efficiency (Fig. 3c)

					P-value
Name	Loop length	Mean	SEM	N value	Template
Control		1.000	0.113	10	
111-T	3	0.150	0.077	3	
cMyc-T	4	0.055	0.023	3	
222-T	6	0.134	0.048	3	
133-T	7	0.120	0.033	3	
333-T	9	0.130	0.027	3	
TTA-T	9	0.264	0.072	3	
AAA-T	9	0.073	0.078	3	
444-T	12	0.109	0.081	3	
555-T	15	0.147	0.046	3	
177-T	15	0.103	0.013	3	
199-T	19	0.238	0.059	3	
111-NT	3	1.001	0.365	3	0.0295
cMyc-NT	4	1.377	0.207	5	0.0001
222-NT	6	1.170	0.433	3	0.0309
133-NT	7	1.851	0.317	3	0.0060

333-NT	9	1.367	0.454	3	0.0248
TTA-NT	9	1.425	0.257	3	0.0062
AAA-NT	9	1.400	0.199	3	0.0041
444-NT	12	1.596	0.237	3	0.0050
555-NT	15	2.253	0.267	3	0.0025
177-NT	15	2.175	0.642	3	0.0181
199-NT	19	3.355	0.476	5	0.0001

*N value means the number of independent events.
*SEM is presented as 95% confidence limit.
**P*-values are reported by two-sided unpaired t-test.

Supplementary Table 3.4: Normalized transcription rate (Fig. 5c)

				P-value
Name	Mean	SEM	N value	Control
Control	1.00	0.08	3	
Non-template	1.13	0.15	3	0.350
RNA 1	0.86	0.06	3	0.014
RNA 2	1.02	0.09	3	0.636
RNA 3	1.02	0.06	3	0.451
RNA 4	1.10	0.11	3	0.415
RNA 5	0.99	0.12	3	0.716
RNA 6	0.92	0.07	3	0.004

*N value means the number of independent events.
*SEM is presented as 95% confidence limit.
**P*-values are compared to control and reported by two-sided paired t-test.

Supplementary Table 3.5: Normalized translation efficiency (Fig. 5d)

				P-value
Name	Mean	SEM	N value	Non-template
Control	1.00	0.03	3	
Non-template	4.53	0.55	3	
RNA 1	4.34	0.56	3	0.135
RNA 2	3.45	0.33	3	0.021
RNA 3	3.97	0.60	3	0.017
RNA 4	3.60	0.59	3	0.014
RNA 5	1.05	0.20	3	0.004
RNA 6	2.21	0.27	3	0.007

*N value means the number of independent events.
*SEM is presented as 95% confidence limit.
**P*-values are compared to NT and reported by two-sided paired t-test.

Supplementary Table 3.6: Probe intensity (Fig. 5h)

	Name	Mean	SEM	N value	*P*-value Control	*P*-value Template
No heating	Control	11.00	1.30	3		
	Template	11.25	3.17	3	0.852	
	Non-template	11.52	1.16	3		0.854
Heated	Control	17.97	0.58	3		
	Template	18.02	1.19	3	0.963	
	Non-template	17.01	2.52	3		0.337
	Probe only	0.28	0.02	3		

*N value means the number of independent events.

*SEM is presented as 95% confidence limit.

**P*-values are reported by two-sided paired t-test.

Supplementary Table 3.7: RT-PCR (Fig. 6b)

	Name	Mean	SEM	N value	*P*-value Control	*P*-value Template
Primer 1	Control	13.35905	0.171649	3		
	Template	13.39015	0.197186	3	0.883005	
	Non-template	13.40935	0.174694	3	0.828291	0.942195
Primer 2	Control	17.14766	0.073494	3		
	Template	17.06876	0.06515	3	0.290763	
	Non-template	17.25807	0.045814	3	0.292033	0.073694

*N value means the number of independent events.

*SEM is presented as 95% confidence limit.

**P*-values are reported by two-sided paired t-test.

Supplementary Table 3.8: Normalized transcription rate (Fig. 6f)

Name	Mean	SEM	N value	*P*-value 0 nM
0 nM DHX36	1.00	0.12	3	
1 nM	1.13	0.05	3	0.096
2 nM	1.09	0.05	3	0.715
5 nM	1.07	0.04	3	0.826

*N value means the number of independent events.

*SEM is presented as 95% confidence limit.

**P*-values are reported by two-sided paired t-test.

Supplementary Table 3.9: Normalized translation efficiency (Fig. 6g)

Name	Mean	SEM	N value	*P*-value
				0 nM
0 nM DHX36	1.00	0.09	3	
1 nM	0.78	0.00	3	9.35E-05
2 nM	0.67	0.01	3	4.34E-03
5 nM	0.23	0.01	3	2.15E-03

*N value means the number of independent events.

*SEM is presented as 95% confidence limit.

**P*-values are reported by two-sided paired t-test.

Supplementary Table 3.10: Normalized translation efficiency (Fig. 7e)

Name	Mean	SEM	N value	*P*-value
				Template
Control	1.60	0.14	3	
111-T	1.08	0.29	3	
cMyc-T	1.08	0.29	5	
555-T	1.51	0.36	3	
222-T	2.99	1.02	3	
133-T	1.03	0.21	3	
TTA-T	1.69	0.56	3	
199-T	1.55	0.42	3	
111-NT	1.89	0.79	3	0.248
cMyc-NT	3.09	0.84	3	0.034
555-NT	4.04	0.93	3	0.024
222-NT	4.39	1.05	3	0.246
133-NT	4.50	1.02	3	0.009
TTA-NT	4.69	1.21	3	0.033
199-NT	5.75	1.43	3	0.016

*N value means the number of independent events.

*SEM is presented as 95% confidence limit.

**P*-values are reported by two-sided paired t-test.

Supplementary Table 3.11: Normalized Translation efficiency (Supplementary Fig. 3b)

Name	Mean	SEM	N value	*P*-value
				cMyc-NT
10hp_Control	1.00	0.03		
10hp_cMyc-NT	5.66	0.24	3	
10hp + hp6	6.34	0.87	3	0.569
10hp + hp10	6.65	0.61	3	0.287
10hp + hp14	6.98	0.52	3	0.132
10hp + hp17	6.87	1.06	3	0.413

*N value means the number of independent events.

*SEM is presented as 95% confidence limit.

**P*-values are reported by two-sided paired t-test.

Supplementary Table 3.12: *E. coli* RT-PCR (Supplementary Fig. 5d)

Name	Mean	SEM	N value	*P*-value	
				Control	199
Control	1.49	0.18	3		
cMyc-T	1.46	0.12	3	0.914	0.042
199-T	1.16	0.09	3	0.236	
cMyc-NT	1.41	0.14	3	0.609	0.475
199-NT	1.33	0.17	3	0.103	

*N value means the number of independent events.

*SEM is presented as 95% confidence limit.

**P*-values are reported by two-sided paired t-test.

Supplementary Table 3.13: Normalized translation efficiency for 0hp-GAA mutants (Supplementary Fig. 6a)

Name	Mean	SEM	N value	*P*-value
				cMyc-NT
0hp_control	2.87	0.56	3	
0hp_cMyc-NT	4.05	0.30	3	
0hp_GAA1	3.31	0.41	3	0.023
0hp_GAA2	5.04	0.91	3	0.251
0hp_GAA3	5.71	1.08	3	0.172
0hp_GAA4	4.82	0.68	3	0.182

*N value means the number of independent events.

*SEM is presented as 95% confidence limit.

**P*-values are reported by two-sided paired t-test.

Supplementary Table 3.14: Normalized translation efficiency for 0hp-GAG mutants (Supplementary Fig. 6b)

Name	Mean	SEM	N value	*P*-value
				cMyc-NT
0hp_control	2.65	0.09	3	
0hp_cMyc-NT	4.04	0.38	3	
0hp_GAG1	2.90	0.19	3	0.064
0hp_GAG12	3.77	0.11	3	0.616
0hp_GAG123	5.07	0.50	3	0.012
0hp_GAG1234	5.99	0.44	3	0.005

*N value means the number of independent events.

*SEM is presented as 95% confidence limit.

**P*-values are reported by two-sided paired t-test.

Supplementary Table 3.15: Normalized Translation efficiency for 10hp-GAG mutants (Supplementary Fig. 6c)

Name	Mean	SEM	N value	*P*-value
				cMyc-NT
10hp_control	1.00	0.03		
10hp_cMyc-NT	4.79	0.74	3	
10hp_GAG1	4.46	0.81	3	0.133
10hp_GAG12	5.62	0.91	3	0.046
10hp_GAG123	5.35	0.90	3	0.093
0hp_GAG1234	6.63	0.73	3	0.001

*N value means the number of independent events.

*SEM is presented as 95% confidence limit.

**P*-values are reported by two-sided paired t-test.

Supplementary Table 3.16: Normalized expression intensity (Supplementary Fig. 7d)

	Name	Mean	SEM	N value	*P*-value
					Template
Negative control		1.00	0.00	3	
Template	111	2.30	0.30	3	
	cMyc	1.94	0.16	5	
	133	1.84	0.16	3	
	222	3.03	0.42	3	
	233	1.90	0.24	3	
	333	2.97	0.43	3	
	TTA	2.86	0.42	3	
	AAA	3.00	0.27	3	

	555	2.21	0.30	3	
Non-template	111	8.84	1.08	3	0.021
	cMyc	9.36	0.88	3	0.014
	133	9.89	0.97	3	0.015
	222	9.39	1.30	3	0.028
	233	8.72	0.87	3	0.013
	333	7.64	1.00	3	0.026
	TTA	10.56	0.86	3	0.006
	AAA	6.15	0.44	3	0.006
	555	13.46	1.40	3	0.015

*N value means the number of independent events.

*SEM is presented as 95% confidence limit.

**P*-values are reported by two-sided paired t-test.
